# 维奈克拉联合酪氨酸激酶抑制剂及减低剂量化疗治疗微小残留病阳性及难治复发Ph阳性急性淋巴细胞白血病13例疗效及安全性分析

**DOI:** 10.3760/cma.j.cn121090-20241202-00521

**Published:** 2025-07

**Authors:** 昊 艾, 桃桃 梁, 倩 王, 鸿飞 吴, 青松 尹

**Affiliations:** 郑州大学附属肿瘤医院、河南省肿瘤医院血液科，郑州 450008 Department of Hematology, the Affiliated Cancer Hospital of Zhengzhou University, Zhengzhou 450008, China

## Abstract

为探讨维奈克拉（Venetoclax，Ven）联合酪氨酸激酶抑制剂（TKI）及减低剂量化疗治疗微小残留病（MRD）阳性及难治复发（R/R）Ph阳性急性淋巴细胞白血病（Ph^+^ ALL）患者的疗效及安全性，回顾性分析2015年7月至2024年2月郑州大学附属肿瘤医院收治的13例MRD阳性及R/R Ph^+^ ALL临床资料，男7例，女6例，中位年龄50（37～71）岁，再诱导治疗采用以Ven、TKI为基础联合减低剂量化疗。13例患者中，MRD阳性10例，R/R 3例。10例MRD阳性患者，9例达分子学缓解（CMR），中位达到时间为47（30～80）d，1例无效；3例R/R患者，2例达完全缓解（CR），1例无效。所有患者中位总生存（OS）及无事件生存（EFS）期分别为21.5个月及7个月；获得CMR患者中位OS及EFS期分别为35个月及34个月。5例患者发生了3级及以上血液学不良反应，所有患者经治疗后造血功能恢复，同时无重症感染及3级以上脏器不良反应发生。提示Ven联合TKI及减低剂量化疗治疗MRD阳性及R/R Ph^+^ ALL，能够明显提高MRD清除率，获得CMR且治疗相关不良反应可耐受。

## Abstract (en)

This study sought to evaluate the efficacy and safety of venetoclax（Ven）in combination with tyrosine kinase inhibitors（TKI）and reduced-dose chemotherapy for the treatment of patients with minimal residual disease（MRD）-positive and relapsed/refractory（R/R）Ph-positive acute lymphoblastic leukemia（Ph^+^ ALL）. A retrospective analysis was conducted on the clinical data of 13 patients with MRD-positive and relapsed Ph^+^ ALL admitted between July 2015 and February 2024 at the Affiliated Cancer Hospital of Zhengzhou University. The cohort included seven males and six females, with a median age of 50 years（range: 37–71 years）. Reinduction therapy consisted of Ven and TKI administration combined with reduced-dose chemotherapy. Among the 13 patients, 10 were MRD-positive, and three had R/R disease. Of the MRD-positive group, nine（90％）achieved complete molecular response（CMR）, with a median time to response of 47 days（range: 30–80）; one patient did not respond. Among the three patients who had R/R, two（66.6％）achieved complete remission, while one patient was nonresponsive. The median overall survival（OS）and relapse-free survival（RFS）time for the entire cohort were 21.5 months and 7 months, respectively. In patients who achieved CMR, the median OS and RFS time were 35 months and 34 months, respectively. Grade ≥3 hematologic adverse events occurred in five patients（38.4％）; however, hematopoietic function recovered in all cases, and no grade ≥3 infections or organ-related adverse reactions were observed. These findings suggest that Ven combined with TKI and reduced-dose chemotherapy may be an effective and tolerable therapeutic strategy for MRD-positive and R/R Ph^+^ ALL, particularly in significantly improving MRD clearance rates.

Ph染色体阳性急性淋巴细胞白血病（Ph^+^ ALL）侵袭性高，常规化疗反应不佳，易产生耐药突变，预后差。酪氨酸激酶抑制剂（TKI）有效改善Ph^+^ ALL早期疗效，通过微小残留病（MRD）转阴后序贯异基因造血干细胞移植（allo-HSCT），延长总生存（OS）期[1]–[2]。然而，临床上仍存在许多因无法耐受高强度治疗及TKI耐药突变等因素导致未达分子学缓解（CMR）及复发患者，即使进行挽救性移植，疗效欠佳。目前，针对不同ABL激酶突变位点，选择相应TKI药物及贝林妥欧单抗、CD22单抗、嵌合抗原受体T（CAR-T）细胞等新的细胞免疫治疗使更多患者获益，但由于价格等因素可及性差。维奈克拉（Ven）直接与Bcl-2蛋白结合，激活线粒体通路诱导细胞凋亡，但却代偿性增加Bim与Mcl-1结合，导致抗凋亡蛋白Mcl-1上调；而TKI可通过下调抗凋亡蛋白Mcl-1表达，抑制白血病细胞增殖并诱导凋亡，两者结合在体外实验及临床小样本应用中展现出良好抗白血病活性[3]。本研究尝试采用Ven联合TKI及减低剂量化疗治疗13例MRD阳性及复发难治（R/R）Ph^+^ ALL患者，观察其疗效及安全性。

## 病例与方法

1. 病例资料：本研究回顾性分析2015年7月至2024年2月郑州大学附属肿瘤医院收治的13例MRD阳性及R/R Ph^+^ ALL患者的临床资料，所有患者参照WHO 2022（第5版）标准，根据细胞形态学、免疫学、细胞遗传学、分子生物学（MICM）分型确诊。以美国东部肿瘤协作组（ECOG）标准进行体能评分。纳入标准：明确诊断为Ph^+^ B-ALL，且满足下列任意一种条件者：①MRD阳性：诱导治疗达完全缓解（CR），MRD持续3个月仍阳性患者或化疗达CMR后MRD转阳患者。②复发难治（R/R）：经规范治疗，首次缓解后12个月内复发；诱导治疗1个疗程未缓解、诱导治疗2个疗程或以上CR后复发、化疗后首次复发再次接受过至少1个疗程挽救治疗后未缓解、HSCT后复发。所有患者均采用Ven联合TKI及减低剂量化疗再诱导治疗。

2. 治疗方案：再诱导方案以Ven联合TKI及减低剂量化疗方案，Ven 100 mg第1天，200 mg第2天，300 mg第3天，400 mg第4～28天，口服，每日1次（Ven用量根据用药期间耐受性、合并用药及骨髓抑制情况调整）；联合治疗方案包括联合氟马替尼+POMP、帕纳替尼+POMP、帕纳替尼+VD、奥雷巴替尼+VID、奥雷巴替尼+减低剂量Hyper-CVAD、奥雷巴替尼。TKI具体用法：氟马替尼：600 mg/d，口服；帕纳替尼：45 mg/d，口服；奥雷巴替尼：40 mg，隔天1次，口服。化疗方案具体用法：POMP方案：巯嘌呤（6-MP）50 mg/m^2^第22～28天，口服；长春新碱（VCR）2 mg第22天，静脉滴注；甲氨蝶呤（MTX）20 mg/m^2^第1、8、15天，口服；地塞米松（DXM）6 mg/m^2^第1～14天，口服。VD方案：VCR 2 mg第1、8天，静脉滴注；DXM 6 mg/m^2^第1～14天，口服。VID方案：VCR 2 mg第1、8天，静脉滴注；伊达比星3 mg/m^2^第1天，静脉滴注；DXM 6 mg/m^2^第1～14天，口服。减低剂量Hyper-CVAD：A方案：CTX 150 mg/m^2^间隔12 h，第1～3天，静脉滴注；VCR 2 mg第1、8天，静脉滴注；DXM针剂20 mg第1～4，11～14天。B方案：MTX 250 mg/m^2^第1天，静脉滴注；阿糖胞苷（Ara-C）0.5 g/m^2^间隔12 h，第2～3天，静脉滴注。采用伏立康唑及泊沙康唑预防及治疗真菌感染时，及时调整Ven及TKI用药剂量。若出现Ⅳ度骨髓抑制，同时合并重症感染时，可停用相应治疗。治疗第21天左右复查骨髓，若MRD呈下降趋势或原始细胞<5％，继续原方案治疗；若下降比例>50％但未达<5％，继续原方案治疗，同时有检测条件的单位应检测Ven或TKI血药浓度；若无效，更改方案再诱导治疗。达CMR或CR后行巩固治疗，巩固治疗仍采用Ven+TKI联合治疗（包括联合POMP、Hyper-CVADA/B、VD、贝林妥欧单抗等）；有合适供者且符合要求者行allo-HSCT/自体造血干细胞移植（auto-HSCT）。

3. 观察指标及治疗期间不良事件的发生及处理原则：治疗前行骨髓穿刺完善MICM分型，化疗结束后复查骨髓涂片及MRD评估疗效。按照NCI常规不良事件标准4.0分级标准评定不良反应。治疗期间定期监测血常规和肝肾功能，针对药物引起的骨髓抑制，采取输注相应血制品及使用促造血生成药物。当患者出现发热或寒战合并感染时，常规进行血培养及病毒检测，并按经验予以广谱抗生素治疗，同时予以泊沙康唑（5 ml/次，每日4次，间隔6 h）预防真菌感染，常规抗生素治疗5～7 d，无效或者CT提示真菌感染者给予静脉抗真菌治疗。

4. 疗效评价及随访：研究主要终点：CMR率及R/R患者CR率。次要终点：OS及无事件生存（EFS）。诱导治疗1个周期进行疗效评估，疗效分为CMR、CR、部分缓解（PR）和疾病进展（PD），存活病例随访至2024年11月15日，死亡病例随访至死亡日期。随访方式包括电话随访、门诊复查、查阅病历，所有患者均未失访。

5. 统计学处理：应用SPSS 23.0软件进行统计学分析。计数资料以例数表示；采用swimmer plots绘制所有患者疗效情况。

## 结果

1. 患者基本临床资料：13例MRD阳性及R/R Ph^+^ALL患者，其中男7例，女6例，中位年龄50（37～71）岁。MRD阳性10例，R/R 3例。10例MRD阳性患者既往化疗中位疗程6（2～19）个；3例R/R患者既往化疗中位疗程7（6～8）个。ABL激酶突变中，T315I 5例，E255K 1例。所有患者临床特征、既往用药及基因突变临床资料详见表1。

**表1:** 13例MRD阳性及复发难治（R/R）Ph^+^急性淋巴细胞白血病患者临床资料

例号	性别	年龄（岁）	诊断	治疗前肿瘤负荷	基因突变	既往化疗方案及线数	既往TKI应用	再诱导方案
1	男	63	MRD+	BM：未见原幼淋 P190：0.024％ MFC-MRD：阴性	阴性	19个疗程后，多次MRD转阳；三线治疗	伊马替尼、达沙替尼	Ven+氟马替尼+改良POMP
2	男	48	MRD+	BM：未见原幼淋 P210：0.037％ MFC-MRD：阴性	TP53	第2疗程缓解，9个疗程后MRD持续阳性；三线治疗	伊马替尼、达沙替尼	Ven+氟马替尼+改良POMP
3	男	69	MRD+	BM：未见原幼淋 P210：1.920％ MFC-MRD：阴性	T315I	第2疗程复发，3个疗程CR后；挽救治疗	伊马替尼、达沙替尼	Ven+帕纳替尼+VD
4	女	57	MRD+	BM：未见原幼淋 P190：0.130％ MFC-MRD：阴性	T315I、IKZF1	第1～3疗程均未CR，第4疗程达CR后；挽救治疗	达沙替尼	Ven+帕纳替尼+改良POMP
5	女	60	MRD+	BM：未见原幼淋 P190：0.169％ MFC-MRD：阴性	E255K	第2疗程复发，4个疗程后达CR后；挽救治疗	伊马替尼、达沙替尼	Ven+氟马替尼+改良POMP
6	女	50	MRD+	BM：未见原幼淋胞 P210：0.016％ MFC-MRD：阴性	IKZF1缺失	4个疗程MRD未转阴	伊马替尼	Ven+氟马替尼+改良POMP
7	男	40	MRD+	BM：未见原幼淋 P210：0.033％ MFC-MRD：阴性	KDM6A、MED2	第1疗程CR，4个疗程后MRD持续阳性	奥雷巴替尼	Ven+奥雷巴替尼+减低剂量HyperCVAD A，B
8	男	37	MRD+	BM：未见原幼淋 P210：0.12％ MFC-MRD：阴性	IKZF1缺失	第1疗程CR，4个疗程后MRD持续阳性	氟马替尼	Ven+奥雷巴替尼+减低剂量HyperCVAD A，B
9	男	58	MRD+	BM：未见原幼淋 P210：0.05％ MFC-MRD：阴性	MED12、RHOA	第1疗程CR，2个疗程后MRD持续阳性	氟马替尼	Ven+奥雷巴替尼+减低剂量HyperCVAD A，B
10	女	55	MRD+	BM：未见原幼淋 P190：0.014％ MFC-MRD：阴性		第1疗程CR，5个周期氟马替尼联合减低剂量化疗及贝林妥欧后MRD持续阳性且比例增高	氟马替尼	Ven+奥雷巴替尼+减低剂量HyperCVAD A，B
11	女	71	R/R	BM：原幼淋占8％ P210：23.7％	T315I、SETD2、IKZF1、PDGFRB	第1疗程CRi，7个疗程后MRD持续阳性	达沙替尼、奥雷巴替尼	Ven+奥雷巴替尼
12	女	45	R/R	BM：原幼淋占84％ P190：65.1％	T315I	第1～3疗程均未CR，MRD持续阳性，6个疗程后复发，采用奥雷巴替尼单药联合化疗未达CR	伊马替尼、氟马替尼、达沙替尼、奥雷巴替尼	Ven+奥雷巴替尼+VID
13	男	47	R/R	BM：原幼淋占82.5％ P190：65.1％	T315I、DNMT3A、NOTCHI	第1疗程达CR后未正规治疗复发，予以达沙替尼、奥雷巴替尼联合化疗未缓解	伊马替尼、达沙替尼、奥雷巴替尼	Ven+奥雷巴替尼+VID

**注** MRD：微小残留病；TKI：酪氨酸激酶抑制剂；原幼淋：原始幼稚淋巴细胞；BM：骨髓；MFC-MRD：流式细胞术检测的微小残留病；CR：完全缓解；Ven：维奈克拉；POMP：巯嘌呤+长春新碱（VCR）+甲氨蝶呤（MTX）+地塞米松（DXM）；VD：VCR+DXM；VID：VCR+伊达比星+DXM；Hyper-CVAD A方案：环磷酰胺+VCR+DXM针剂；Hyper-CVAD B方案：MTX+阿糖胞苷

2. 疗效评价：所有患者均至少完成1个疗程联合治疗，可评估疗效。10例MRD阳性患者中，9例疗效评价为CMR，中位达到时间为47（30～80）d，2例达CMR后序贯allo-HSCT。3例R/R患者中，2例疗效评价为CR，1例疗效评价为PD。5例T315I突变患者中，2例为MRD阳性患者，1例疗效评价为CMR，1例疗效评价为PD；3例为R/R患者，2例疗效评价为CR，1例疗效评价为PD。具体疗效评估见图1。

**图1:**
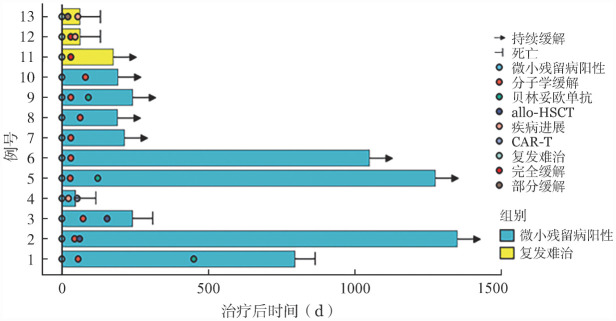
维奈克拉联合酪氨酸激酶抑制剂及减低剂量化疗治疗13例微小残留病阳性及复发Ph阳性急性淋巴细胞白血病患者的疗效及生存情况泳道图

3. 长期疗效及生存情况：截至随访终点，所有患者中位随访时间为15（1～45）个月，随访期内，中位OS期及EFS期分别为21.5个月及7个月。达CMR患者中位OS期及EFS期分别为35个月及34个月。

MRD阳性患者中，例1因甲流性肺炎死亡；例3（T315I突变患者）达CMR后序贯allo-HSCT，因急性移植物抗宿主病（GVHD）合并重症肺炎死亡；例4（T315I突变患者）PD后序贯CAR-T细胞治疗，因疾病进展死亡。其余7例患者无复发生存至今。

3例R/R患者均为T315I突变患者，例12达CR后再次PD，因PD死亡；例13无效，因PD死亡；例11达CR后采用Ven联合TKI+POMP方案维持无复发生存至今。

例1、5、9达CMR后，间断予以贝林妥欧单抗，均无复发生存至今。例2达CMR后序贯allo-HSCT，无复发生存至今；例3达CMR后序贯allo-HSCT，因急性GVHD合并重症肺炎死亡。5例T315I突变患者中，3例因PD死亡，1例因合并急性GVHD、重症感染死亡，仅1例无复发生存至今。具体生存情况见图1。

4. 不良反应：主要为Ⅰ～Ⅱ度骨髓抑制，仅有4例患者出现Ⅲ～Ⅳ度骨髓抑制，予以相应对症处理10 d左右好转。部分患者出现轻度皮肤色素沉着、胃肠道反应和乏力，患者均可耐受，经处理后可缓解。未见胸水、血栓、心律失常、水肿、肌肉关节疼痛及肺部重症感染等不良事件，无治疗相关的死亡。

## 讨论

Ph^+^ ALL侵袭性强，预后不佳。研究显示在未进行allo-HSCT情况下，Ph^+^ ALL中位无病生存（DFS）期及OS期分别为6个月及9个月[4]。常规治疗采用TKI联合中小剂量化疗，3个月内获得CMR预后良好，首次CR后进行allo-HSCT可改善预后[5]；我中心前期回顾性分析一、二代TKI联合化疗治疗Ph^+^ ALL疗效比较提示，是否获得CMR对OS及RFS至关重要[6]。因此，allo-HSCT虽被认为是Ph^+^ ALL长生存的唯一选择[7]，但缓解深度直接影响长期预后。随着TKI、贝林妥欧单抗、CD22单抗、CAR-T等细胞免疫靶向治疗策略应用于临床，使得Ph^+^ ALL早期疗效得到改善，通过CMR后序贯allo-HSCT/auto-HSCT，提高OS及DFS率[1]–[2],[8]。然而，临床上仍存在无法耐受联合密集治疗、TKI耐药突变、细胞免疫治疗不可及等因素导致MRD增高及R/R Ph^+^ ALL。因此，选择何种药物及方案与TKI协同，以达到CMR值得探讨。

TKI药物耐机制可能为白血病细胞通过ABL激酶结构域突变及增加不涉及ABL信号平行通路的旁路信号激活等方式发生作用，最终通过上调Bcl-2家族抗凋亡蛋白表达，避免细胞凋亡，提高存活率[9]。有研究表明多种淋巴造血系统恶性疾病的发生、发展与抗凋亡蛋白Bcl-2及促凋亡蛋白Bim/Bax等相关。因此，针对抑制Bcl-2蛋白表达靶向治疗成为强有力选择靶点[10]。尽管部分体外实验表明Ph^+^ ALL细胞表面Bcl-2蛋白表达量相对较低，但仍能被RNA靶向干扰，促进细胞系凋亡[11]。

Ven通过抑制抗凋亡蛋白Bcl-2，激活线粒体通路，诱导细胞凋亡。目前在包括慢性淋巴细胞白血病、急性髓系白血病、非霍奇金淋巴瘤及MLL阳性ALL等治疗中体现较强活性[12]–[13]。Ven通过选择性抑制Bcl-2而非Mcl-1途径调控细胞凋亡；Ven虽减少了Bim与Bcl-2的结合，但代偿性增加Bim与Mcl-1结合，从而导致抗凋亡蛋白Mcl-1上调，而Mcl-1作为人髓系白血病细胞系ML-1分化过程中发现的早期诱导基因，通过捕获Bim，使Bim无法活化Bax/Bak，从而抑制Bcl-2抑制剂诱导细胞凋亡，导致细胞凋亡的降低。与此同时，有研究发现Ph^+^ ALL小鼠模型中异常Mcl-1蛋白表达与白血病发生、发展相关，选择性抑制Mcl-1表达及活性具有潜在治疗价值。TKI可下调抗凋亡蛋白Mcl-1表达，抑制白血病细胞增殖并诱导凋亡，从而得出：Bcl-2抑制剂联合Mcl-1抑制剂，通过下调Mcl-1途径与Ven联合应用在Ph^+^ ALL细胞系表现出强大的协同作用[3]。此外，临床前研究也显示Ven、TKI及糖皮质激素三药联合诱导酪氨酸激酶LYN上调，促进促凋亡Bim和抑制抗凋亡蛋白Mcl-1表达，诱导细胞凋亡，在抗肿瘤和生存获益方面优于两药联合[14]–[15]。国外多项小样本临床研究均显示Ven联合TKI及小剂量化疗疗效尚可[16]–[17]。本研究回顾分析了13例接受Ven联合TKI及减低剂量化疗治疗MRD阳性及R/R-Ph^+^ ALL患者疗效及安全性。对于MRD阳性患者，90％（9/10）达CMR；对于R/R患者，66.6％（2/3）达CR。获得CMR患者通过Ven+TKI联合POMP、Hyper-CVADA/B、VD、贝林妥欧单抗、allo-HSCT等巩固强化方式获得更长OS及EFS期。合并T315I突变5例患者整体预后差，即使选择此方案也仅取得短期疗效，仅1例达CR后原方案维持至今，1例获得CMR序贯allo-HSCT，因感染死亡，余3例均因疾病进展死亡。安全性方面，仅5例（38.4％）患者发生了3级及以上血液学不良反应，所有患者经治疗后造血功能恢复，未出现重症感染及3级以上脏器不良反应。

综上所述，Ven联合TKI及减低剂量化疗方案治疗总体耐受性良好，高效低毒，尤其对于MRD阳性患者，提高了CMR率及长期无复发生存率，展现出了一定优势，并为移植前高效清除MRD提供相对高性价比方案。但由于是回顾性研究，样本量较小，且随访时间短，结果会存在一定片面性，仍需后期扩大样本及延长随访时间，从而获得更确切的结论。
